# Supplementary material for: Emissions reduction and pricing of supply chain under cap-and-trade and subsidy mechanisms
Source: PLoS One. 2022 Apr 1;17(4):e0266413. doi: 10.1371/journal.pone.0266413 (PMC8975140; doi:10.1371/journal.pone.0266413)
Supplement: S1 Appendix — (DOCX) [file pone.0266413.s001.docx]

**Appendix A**

Proof of Theorem 1:

$\pi_{m}^{M}=\left( w_{1}-c_{1} \right)Q_{1}+\left( w_{2}{-c}_{2}+s \right)Q_{2}-\frac{1}{2}\varepsilon e^{2}-p_{c}\left[ e_{1}Q_{1}+\left( e_{1}-e \right)Q_{2}-E_{g} \right]$ （1）

$\pi_{r}^{M}=\left( p_{1}-w_{1} \right)Q_{1}+\left( p_{2}{-w}_{2} \right)Q_{2}$（2）

Its corresponding Hessian matrix is $H_{(\pi_{r}^{M})}=\left| \begin{matrix} \frac{{\partial^{2}}_{\pi_{r}^{M}}}{\partial p_{1}^{2}} & \frac{\partial^{2}\pi_{r}^{M}}{\partial p_{1}\partial p_{2}} \\ \frac{\partial^{2}\pi_{r}^{M}}{\partial p_{2}\partial p_{1}} & \frac{\partial^{2}\pi_{r}^{M}}{\partial p_{2}^{2}} \end{matrix} \right|=\left| \begin{matrix} -2 & 1 \\ 1 & -2 \end{matrix} \right|$，

Given $\left| H_{(\pi_{r}^{M})} \right|=\left( -2 \right)^{2}-1^{2}=3>0$ and $-2<0, H_{(\pi_{r}^{M})}$ is a negative definite matrix and $\pi_{r}^{M}$ has a local maximum value. Calculate the partial derivatives of retail prices $p_{1}$ and $p_{2}$, and let these partial derivatives equal to 0; thus, we can find:

$\left\{ \begin{aligned} p_{1}=\frac{w_{1}+w_{2}+1}{3} \\ p_{2}=\frac{2w_{2}-w_{1}+2}{3} \end{aligned} \right.$ （3）

Put Equation (3) into Equation (1), and we can obtain the corresponding Hessian matrix, expressed as:

$H_{(\pi_{m}^{M})}=\left| \begin{matrix} \frac{\partial^{2}\pi_{m}^{M}}{\partial w_{1}^{2}} & \frac{\partial^{2}\pi_{m}^{M}}{\partial w_{1}\partial w_{2}} \\ \frac{\partial^{2}\pi_{m}^{M}}{\partial w_{2}\partial w_{1}} & \frac{\partial^{2}\pi_{m}^{M}}{\partial w_{2}^{2}} \end{matrix} \right|=\left| \begin{matrix} -\frac{4}{3} & \frac{2}{3} \\ \frac{2}{3} & -\frac{4}{3} \end{matrix} \right|$，

Given $\left| H_{(\pi_{m}^{M})} \right|={(-\frac{4}{3})}^{2}-({\frac{2}{3})}^{2}=\frac{4}{3}>0$ and$-\frac{4}{3}<0, H_{(\pi_{m}^{M})}$ represents a negative definite matrix and $\pi_{m}^{M}$ has a local maximum value. Calculate the partial derivatives of $w_{1}、w_{2}$, and let these partial derivatives equal to 0; Thus, we can measure the wholesale price by:

$\left\{ \begin{aligned} w_{1}^{M}=\frac{1+c_{1}+p_{c}e_{1}}{2} \\ w_{2}^{M}=\frac{1+c_{2}-s+p_{c}e_{1}-p_{c}e}{2} \end{aligned} \right.$ （4）

Put Equation (4) into Equation (3), and we can calculate the optimal retail price and sales volume when manufacturers are subsidized:

$\left\{ \begin{aligned} p_{1}^{M}=\frac{4+c_{1}+c_{2}+{2p}_{c}e_{1}-p_{c}e-s}{6} \\ p_{2}^{M}=\frac{5-c_{1}+2c_{2}+p_{c}e_{1}-2p_{c}e-2s}{6} \end{aligned} \right.$ (5)

$\left\{ \begin{aligned} Q_{1}^{M}=\frac{1-2c_{1}+c_{2}-p_{c}e_{1}-p_{c}e-s}{6} \\ Q_{2}^{M}=\frac{1+c_{1}-2c_{2}-p_{c}e_{1}+2p_{c}e+2s}{6} \end{aligned} \right.$ (6)

Put the results into Equation (1), calculate the first derivative of $e$, and let it amount to 0, thus we can obtain:

$e^{M}=\frac{p_{c}^{2}e_{1}+2c_{2}p_{c}-c_{1}p_{c}-p_{c}-2p_{c}s}{2p_{c}^{2}-6\varepsilon}$

**Appendix B**

Proof of Theorem 2:

$\pi_{m}^{R}=\left( w_{1}-c_{1} \right)Q_{1}+\left( w_{2}{-c}_{2} \right)Q_{2}-\frac{1}{2}\varepsilon e^{2}-p_{c}\left[ e_{1}Q_{1}+\left( e_{1}-e \right)Q_{2}-E_{g} \right]$（7）

$\pi_{r}^{R}=\left( p_{1}-w_{1} \right)Q_{1}+\left( p_{2}{-w}_{2}+s \right)Q_{2}$（8）

The Hessian matrix of the retailers' profit function is $H_{(\pi_{r}^{R})}=\left| \begin{matrix} \frac{\partial^{2}\pi_{r}^{R}}{\partial p_{1}^{2}} & \frac{\partial^{2}\pi_{r}^{R}}{\partial p_{1}\partial p_{2}} \\ \frac{\partial^{2}\pi_{r}^{R}}{\partial p_{2}\partial p_{1}} & \frac{\partial^{2}\pi_{r}^{R}}{\partial p_{2}^{2}} \end{matrix} \right|=\left| \begin{matrix} -2 & 1 \\ 1 & -2 \end{matrix} \right|$

Given $\left| H_{(\pi_{r}^{R})} \right|=\left( -2 \right)^{2}-1^{2}=3>0,-2<0, H_{(\pi_{r}^{R})}$ is known as a negative definite matrix and $\pi_{r}^{R}$ has a maximum value. Calculate the partial derivatives of $p_{1}$ and $p_{2}$, and let $\frac{\partial\pi_{r}^{R}}{\partial p_{1}}=0, and \frac{\partial\pi_{r}^{R}}{\partial p_{2}}=0$, thus we measure the retail price by: $\left\{ \begin{aligned} p_{1}=\frac{w_{1}+w_{2}+1-s}{3} \\ p_{2}=\frac{2w_{2}-w_{1}+2-2s}{3} \end{aligned} \right.$（9）

$\left\{ \begin{aligned} Q_{1}=p_{2}-p_{1}=\frac{w_{2}-2w_{1}-3s+1}{3} \\ Q_{2}=1-p_{2}=\frac{w_{1}-2w_{2}+2s+1}{3} \end{aligned} \right.$（10）

Put these equations into Equation (7), and we will obtain $H_{(\pi_{m}^{R})}=\left| \begin{matrix} \frac{\partial^{2}\pi_{m}^{R}}{\partial w_{1}^{2}} & \frac{\partial^{2}\pi_{m}^{R}}{\partial w_{1}\partial w_{2}} \\ \frac{\partial^{2}\pi_{m}^{R}}{\partial w_{2}\partial w_{1}} & \frac{\partial^{2}\pi_{m}^{R}}{\partial w_{2}^{2}} \end{matrix} \right|=\left| \begin{matrix} -\frac{4}{3} & \frac{2}{3} \\ \frac{2}{3} & -\frac{4}{3} \end{matrix} \right|$

Considering $\left| H_{(\pi_{m}^{R})} \right|={(-\frac{4}{3})}^{2}-({\frac{2}{3})}^{2}=\frac{4}{3}>0$ and $-\frac{4}{3}<0, H_{(\pi_{m}^{R})}$ represents a negative definite matrix and $\pi_{m}^{R}$ has a maximum value. The wholesale price obtained is:

$\left\{ \begin{aligned} w_{1}^{R}=\frac{1+c_{1}+p_{c}e_{1}}{2}-\frac{2s}{3} \\ w_{2}^{R}=\frac{1+c_{2}+p_{c}e_{1}-p_{c}e}{2}+\frac{s}{6} \end{aligned} \right.$ . Put them into Equations (9) and (10), respectively, we obtain:

$\left\{ \begin{aligned} p_{1}^{R}=\frac{4+c_{1}+c_{2}+{2p}_{c}e_{1}-p_{c}e-3s}{6} \\ p_{2}^{R}=\frac{5-c_{1}+2c_{2}+p_{c}e_{1}-2p_{c}e-2s}{6} \end{aligned} \right.$ （11）

$\left\{ \begin{aligned} Q_{1}^{R}=\frac{1-2c_{1}+c_{2}-p_{c}e_{1}-p_{c}e+s}{6} \\ Q_{2}^{R}=\frac{1+c_{1}-2c_{2}-p_{c}e_{1}+2p_{c}e+2s}{6} \end{aligned} \right.$ （12）

Put them into Equation (7) and calculate the partial derivative of $e$, we find that:

$e^{R}=\frac{p_{c}^{2}e_{1}+2p_{c}c_{2}-{p_{c}c}_{1}-p_{c}-2p_{c}s}{2p_{c}^{2}-6\varepsilon}$

**Appendix C**

Proof of Theorem 3:

$\pi_{m}^{C}=\left( w_{1}-c_{1} \right)Q_{1}+\left( w_{2}{-c}_{2} \right)Q_{2}-\frac{1}{2}\varepsilon e^{2}-p_{c}\left[ e_{1}Q_{1}+\left( e_{1}-e \right)Q_{2}-E_{g} \right]$ (13)

The function of retailers' profit can be expressed as: $\pi_{r}^{C}=\left( p_{1}-w_{1} \right)Q_{1}+\left( p_{2}{-w}_{2} \right)Q_{2}$(14)

$H_{(\pi_{r}^{C})}=\left| \begin{matrix} \frac{\partial^{2}\pi_{r}^{C}}{\partial p_{1}^{2}} & \frac{\partial^{2}\pi_{r}^{C}}{\partial p_{1}\partial p_{2}} \\ \frac{\partial^{2}\pi_{r}^{C}}{\partial p_{2}\partial p_{1}} & \frac{\partial^{2}\pi_{r}^{C}}{\partial p_{2}^{2}} \end{matrix} \right|=\left| \begin{matrix} -2 & 1 \\ 1 & -2 \end{matrix} \right|$

Considering $\left| H_{(\pi_{r}^{C})} \right|=\left( -2 \right)^{2}-1^{2}=3>0$and $-2<0$，$H_{(\pi_{r}^{C})}$ is a negative definite matrix and $\pi_{r}^{C}$has a maximum value. Calculate the first derivatives of $p_{1}$ and $p_{2}$, and let $\frac{\partial\pi_{r}^{C}}{\partial p_{1}}=0$ and $\frac{\partial\pi_{r}^{C}}{\partial p_{2}}=0$, we can obtain the retail price and sales volume:

$\left\{ \begin{aligned} p_{1}=\frac{w_{1}+w_{2}+1-s}{3} \\ p_{2}=\frac{2w_{2}-w_{1}+2+s}{3} \end{aligned} \right.$（15）

$\left\{ \begin{aligned} Q_{1}=p_{2}-s-p_{1}=\frac{w_{2}-2w_{1}+1-s}{3} \\ Q_{2}=1-p_{2}+s=\frac{w_{1}-2w_{2}+1+2s}{3} \end{aligned} \right.$（16）

Put them into Equation (13), we can have a new profit function, whose Hessian matrix is:

$H_{(\pi_{m}^{C})}=\left| \begin{matrix} \frac{\partial^{2}\pi_{m}^{C}}{\partial w_{1}^{2}} & \frac{\partial^{2}\pi_{m}^{C}}{\partial w_{1}\partial w_{2}} \\ \frac{\partial^{2}\pi_{m}^{C}}{\partial w_{2}\partial w_{1}} & \frac{\partial^{2}\pi_{m}^{C}}{\partial w_{2}^{2}} \end{matrix} \right|=\left| \begin{matrix} -\frac{4}{3} & \frac{2}{3} \\ \frac{2}{3} & -\frac{4}{3} \end{matrix} \right|$

Given $\left| H_{(\pi_{m}^{C})} \right|={(-\frac{4}{3})}^{2}-({\frac{2}{3})}^{2}=\frac{4}{3}>0$ and $-\frac{4}{3}<0, H_{(\pi_{m}^{C})}$ represents a negative definite matrix and $\pi_{m}^{C}$ has a local maximum value. Calculate the first derivatives of $w_{1}$ and $w_{2}$ and let these derivatives equal to 0, we can find that:

$\left\{ \begin{aligned} w_{1}^{C}=\frac{1+c_{1}+p_{c}e_{1}}{2} \\ w_{2}^{C}=\frac{1+c_{2}+p_{c}e_{1}-p_{c}e+s}{2} \end{aligned} \right.$

Put it into Equations (15) and (16), and we obtain the retail price and sales volume under the C-mode:

$\left\{ \begin{aligned} p_{1}^{C}=\frac{4+c_{1}+c_{2}+2p_{c}e_{1}-p_{c}e-s}{6} \\ p_{2}^{C}=\frac{5-c_{1}+2c_{2}+p_{c}e_{1}-2p_{c}e+4s}{6} \end{aligned} \right.$

$\left\{ \begin{aligned} Q_{1}^{C}=\frac{1-2c_{1}+c_{2}-p_{c}e_{1}-p_{c}e-s}{6} \\ Q_{2}^{C}=\frac{1+c_{1}-2c_{2}-p_{c}e_{1}+2p_{c}e+2s}{6} \end{aligned} \right.$

Put them into Equation (13) and calculate the partial derivative of $e$ based on the new function of manufacturers' profits, we can obtain the optimal carbon emissions under the C-mode:

$e^{C}=\frac{p_{c}^{2}e_{1}+2p_{c}c_{2}-p_{c}c_{1}-p_{c}-2p_{c}s}{2p_{c}^{2}-6\varepsilon}$

**Appendix D**

Proof of Inference 1:

$e^{M}=e^{R}=e^{C}=\frac{p_{c}^{2}e_{1}+2p_{c}c_{2}-p_{c}c_{1}-p_{c}-2p_{c}s}{2p_{c}^{2}-6\varepsilon}$, the optimal carbon emissions reductions per unit of low-carbon product are the same.

Given $\frac{\partial e^{M}}{\partial\varepsilon}=\frac{\partial e^{R}}{\partial\varepsilon}=\frac{\partial e^{C}}{\partial\varepsilon}=\frac{6(p_{c}^{2}e_{1}-p_{c}+2c_{2}p_{c}-c_{1}p_{c}-2p_{c}s)}{{(2p_{c}^{2}-6\varepsilon)}^{2}}, p_{c}^{2}e_{1}+2p_{c}c_{2}-p_{c}c_{1}-p_{c}-2p_{c}s<0$, thus, $\frac{\partial e^{M}}{\partial\varepsilon}<0$. The optimal carbon emissions reductions per unit of low-carbon product are negatively correlated with the investment cost coefficient.

Considering $\frac{\partial e^{M}}{\partial s}=\frac{\partial e^{R}}{\partial s}=\frac{\partial e^{C}}{\partial s}=\frac{-2p_{c}}{2p_{c}^{2}-6\varepsilon}, 2p_{c}^{2}-6\varepsilon<0$, thus, $\frac{\partial e^{M}}{\partial s}>0$. The optimal carbon emissions reductions per unit of low-carbon product are positively correlated with low-carbon subsidies.

**Appendix E**

Proof of Inference 2:

$w_{2}^{C}>w_{2}^{R}{>w}_{2}^{M}$, $\frac{\partial w_{2}^{M}}{\partial s}=\frac{2p_{c}^{2}-J}{2J}=\frac{3\varepsilon}{J}<0$, $\frac{\partial w_{2}^{R}}{\partial s}=\frac{8p_{c}^{2}-6\varepsilon}{6J}, 2\varepsilon<p_{c}^{2}<3\varepsilon$, thus, $\frac{\partial w_{2}^{R}}{\partial s}<0. \frac{\partial w_{2}^{C}}{\partial s}=\frac{4p_{c}^{2}-6\varepsilon}{2J}<0$.There is a negative correlation between the wholesale price of low-carbon products and low-carbon incentives.

$p_{2}^{C}{>p_{2}^{M}=p}_{2}^{R}, \frac{\partial p_{2}^{M}}{\partial s}=\frac{\partial p_{2}^{R}}{\partial s}=\frac{4p_{c}^{2}-2J}{6J}=\frac{2\varepsilon}{J}<0, \frac{\partial p_{2}^{C}}{\partial s}=\frac{2p_{c}^{2}-4\varepsilon}{J}<0$.The retail prices of low-carbon goods are negatively correlated with low-carbon subsidies.

$Q_{2}^{M}=Q_{2}^{R}=Q_{2}^{C}, \frac{\partial Q_{2}^{M}}{\partial s}=\frac{\partial Q_{2}^{R}}{\partial s}=\frac{\partial Q_{2}^{C}}{\partial s}=-\frac{2\varepsilon}{J}>0$.There is a positive correlation between demand for low-carbon goods and government subsidies.

**Appendix F**

Proof of Inference 3:

$w_{1}^{M}=w_{1}^{C}>w_{1}^{R}, \frac{\partial w_{1}^{R}}{\partial s}=-\frac{2}{3}<0$.

There is a negative correlation between the wholesale price of common products and subsidies under R-mode.

${p_{1}^{M}=p}_{1}^{C}>p_{1}^{R}$,$\frac{\partial p_{1}^{M}}{\partial s}= \frac{\partial p_{1}^{C}}{\partial s}=\frac{2p_{c}^{2}-J}{6J}=\frac{\varepsilon}{J}<0, \frac{\partial p_{1}^{R}}{\partial s}=\frac{-4p_{c}^{2}+18\varepsilon}{6J}=-\frac{2p_{c}^{2}-6\varepsilon-3\varepsilon}{3J}<0$.

There is a negative correlation between the retail price of common products and government subsidies.

${Q_{1}^{R}>Q}_{1}^{M}=Q_{1}^{C}, \frac{\partial Q_{1}^{M}}{\partial s}=\frac{\partial Q_{1}^{C}}{\partial s}=\frac{2p_{c}^{2}-J}{6J}=\frac{\varepsilon}{J}<0, \frac{\partial Q_{1}^{R}}{\partial s}=\frac{4p_{c}^{2}-6\varepsilon}{6J}<0$.The sales volume of common products is negatively correlated with government subsidies.

Appendix G

Proof of inference 4:

$E^{M}=E^{C}=e_{1}Q_{1}+e_{2}Q_{2}=e_{1}\frac{1-2c_{1}+c_{2}-p_{c}e_{1}-p_{c}e-s}{6}+e_{2}Q_{2}$

$E^{R}=e_{1}Q_{1}+e_{2}Q_{2}=e_{1}\frac{1-2c_{1}+c_{2}-p_{c}e_{1}-p_{c}e+s}{6}+e_{2}Q_{2}$

${\Delta E=E}^{R}-E^{M}=\frac{e_{1}s}{3}>0$

$\frac{\partial\Delta E}{\partial s}=\frac{e_{1}}{3}>0$

Appendix H

Proof of inference 5:

$E^{M}=e_{1}\cdot\frac{1-2c_{1}+c_{2}-p_{c}e_{1}-p_{c}e-s}{6}+\left( e_{1}-e \right)\cdot\frac{1+c_{1}-2c_{2}-p_{c}e_{1}+2p_{c}e+2s}{6}$

$E^{R}=e_{1}\cdot\frac{1-2c_{1}+c_{2}-p_{c}e_{1}-p_{c}e+s}{6}+\left( e_{1}-e \right)\cdot\frac{1+c_{1}-2c_{2}-p_{c}e_{1}+2p_{c}e+2s}{6}$

$E^{C}=e_{1}\cdot\frac{1-2c_{1}+c_{2}-p_{c}e_{1}-p_{c}e-s}{6}+\left( e_{1}-e \right)\cdot\frac{1+c_{1}-2c_{2}-p_{c}e_{1}+2p_{c}e+2s}{6}$

Let $1-2c_{1}+c_{2}-p_{c}e_{1}=A$，$1+c_{1}-2c_{2}-p_{c}e_{1}=B$

$E^{M}=E^{C}=e_{1}*\frac{A-p_{c}*\frac{K-2p_{c}s}{J}-s}{6}+\left( e_{1}-\frac{K-2p_{c}s}{J} \right)*\frac{B+2p_{c}*\frac{K-2p_{c}s}{J}+2s}{6}=e_{1}*\frac{A-p_{c}*\frac{K}{J}+\frac{6\varepsilon s}{J}}{6}+\left( e_{1}-\frac{K-2p_{c}s}{J} \right)*\frac{B+2p_{c}*\frac{K}{J}-\frac{12\varepsilon s}{J}}{6}$

$\frac{\partial E^{M}}{\partial s}=\frac{\partial E^{C}}{\partial s}=\frac{e_{1}\varepsilon}{J}+\frac{2p_{c}}{J}*\frac{B+2p_{c}*\frac{K}{J}-\frac{12\varepsilon s}{J}}{6}-\frac{e_{1}J-K+2p_{c}s}{J}*\frac{2\varepsilon}{J}=\frac{2p_{c}BJ+4p_{c}^{2}K+12k\varepsilon-6e_{1}\varepsilon J-48p_{c}\varepsilon s}{6J^{2}}$

Considering $\frac{\partial^{2}E^{M}}{\partial s^{2}}=\frac{\partial^{2}E^{C}}{\partial s^{2}}=\frac{-8p_{c}\varepsilon}{J^{2}}<0$, the total carbon emissions represent a concave function with a maximum value. Assuming $\frac{2p_{c}BJ+4p_{c}^{2}K+12k\varepsilon-6e_{1}\varepsilon J}{48p_{c}\varepsilon}>0$, when $s=\frac{2p_{c}BJ+4p_{c}^{2}K+12k\varepsilon-6e_{1}\varepsilon J}{48p_{c}\varepsilon}$, the values of $E^{M}$ and $E^{C}$ are the greatest; when $s^{M}=s^{C}>\frac{2p_{c}BJ+4p_{c}^{2}K+12k\varepsilon-6e_{1}\varepsilon J}{48p_{c}\varepsilon}$, carbon emissions fall as subsidies increase.

$E^{R}=e_{1}*\frac{A-p_{c}*\frac{K-2p_{c}s}{J}+s}{6}+\left( e_{1}-\frac{K-2p_{c}s}{J} \right)*\frac{B+2p_{c}*\frac{K-2p_{c}s}{J}+2s}{6}=e_{1}*\frac{A-p_{c}*\frac{K}{J}+\frac{\left( 4p_{c}^{2}-6\varepsilon\right)s}{J}}{6}+\left( e_{1}-\frac{K-2p_{c}s}{J} \right)*\frac{B+2p_{c}*\frac{K}{J}-\frac{12\varepsilon s}{J}}{6}$

$\frac{\partial E^{R}}{\partial s}=\frac{e_{1}\left( 4p_{c}^{2}-6\varepsilon\right)}{6J}+\frac{2p_{c}}{J}*\frac{B+2p_{c}*\frac{K}{J}-\frac{12\varepsilon s}{J}}{6}-\frac{e_{1}J-K+2p_{c}s}{J}*\frac{2\varepsilon}{J}=\frac{2p_{c}BJ+4p_{c}^{2}K+12k\varepsilon+4p_{c}^{2}e_{1}J-18e_{1}\varepsilon J-48p_{c}\varepsilon s}{6J^{2}}$

$\frac{\partial^{2}E^{R}}{\partial s^{2}}=\frac{-8p_{c}}{J^{2}}<0$

$s^{R}=\frac{2p_{c}BJ+4p_{c}^{2}K+12k\varepsilon+4p_{c}^{2}e_{1}J-18e_{1}\varepsilon J}{48p_{c}\varepsilon}$

$s^{R}-s^{M}=\frac{4p_{c}^{2}e_{1}J-12e_{1}\varepsilon J}{48p_{c}\varepsilon}=\frac{\left( 2p_{c}^{2}-6\varepsilon\right)e_{1}J}{24p_{c}\varepsilon}=\frac{e_{1}J^{2}}{24p_{c}\varepsilon}>0$
