# Supplementary material for: Emissions reduction and pricing of supply chain under cap-and-trade and subsidy mechanisms
Source: PLoS One. 2022 Apr 1;17(4):e0266413. doi: 10.1371/journal.pone.0266413 (PMC8975140; doi:10.1371/journal.pone.0266413)
Supplement: Attachment — Submitted filename: Editor letter.pdf [file pone.0266413.s002.pdf]

Despite my repeated efforts to solicit more reviews, I was only able to secure one reviewer. Fortunately, I am interested myself and somewhat familiar with this line of work, and I read the paper closely myself. Overall I have a quite favorable opinion of the paper. I think the topic is quite interesting and timely; the model is relatively clean and organized; and it is great idea to compare different modes; and lastly, the analysis seems competently done. With all that said, the current draft of the paper also has some problems. Therefore, I would like to invite the authors to revise the paper, addressing my and the reviewer's comments. I deem this revision as in between major and minor revision.

1. On p. 3, row 103, you present these as "hypotheses". Hypotheses are things which you later test whether they hold or not. Rather, the correct term in your setting seems "assumptions". You are merely imposing assumptions for the model.

2. Same page, a few rows down (row 107): the demand for low-carbon good is Q2=1-p2. I understand that this is probably needed for the analysis to be tractable. But this is such a strong assumption, and may drive your qualitative results, you should spend sometime motivating/discussing this assumption.

3. When comparing different modes, I am not sure whether you can or should use the same subsidy "s". There are various reasons why you shouldn't. For one, the subsidy is not absolute amount, but rather rate (per unit subsidy). Since the amount of output differs across modes, the total subsidy amounts will differ as well. I would thus suggest using different notations: s_M, s_R and s_C. The question then is how to compare across modes. One possibility is to restrict total subsidy amount to be the same across modes. The other is to add a stage where the government chooses "s" under each mode to maximize its objective function (social surplus?).

4. Presentation of results

- I would suggest that you report the equilibrium expressions of some key variables, such as emission, profit, consumer surplus, social surplus, government expenditure etc.
- At the same time, relegate some the detailed steps to the Appendix.
- Right now, you report too many results (as Inferences). I suggest that you focus on what you think are the most important results, combine them into fewer propositions, and leave the detailed proofs in the Appendix.

5. On p. 8, row 240, did you verify that $Q_1 \geq 0$ is satisfied in the equilibrium?
